# Assembly of Peptidoglycan Fragments—A Synthetic Challenge

**DOI:** 10.3390/ph13110392

**Published:** 2020-11-15

**Authors:** Fausto Queda, Gonçalo Covas, Sérgio R. Filipe, M. Manuel B. Marques

**Affiliations:** 1 LAQV@REQUIMTE, Departamento de Química, Faculdade de Ciências e Tecnologia, Universidade Nova de Lisboa, Campus de Caparica, 2829-516 Caparica, Portugal; f.queda@campus.fct.unl.pt; 2 UCIBIO@REQUIMTE, Departamento de Ciências da Vida, Faculdade de Ciências e Tecnologia, Universidade Nova de Lisboa, Campus de Caparica, 2829-516 Caparica, Portugal; goncalocovas@itqb.unl.pt; 3 Laboratory of Bacterial Cell Surfaces and Pathogenesis, Instituto de Tecnologia Química e Biológica, Universidade Nova de Lisboa, 2780-157 Oeiras, Portugal

**Keywords:** bacterial peptidoglycan, PGN biosynthesis, PGN synthesis, NAG-NAM disaccharide

## Abstract

Peptidoglycan (PGN) is a major constituent of most bacterial cell walls that is recognized as a primary target of the innate immune system. The availability of pure PGN molecules has become key to different biological studies. This review aims to (1) provide an overview of PGN biosynthesis, focusing on the main biosynthetic intermediates; (2) focus on the challenges for chemical synthesis posed by the unique and complex structure of PGN; and (3) cover the synthetic routes of PGN fragments developed to date. The key difficulties in the synthesis of PGN molecules mainly involve stereoselective glycosylation involving NAG derivatives. The complex synthesis of the carbohydrate backbone commonly involves multistep sequences of chemical reactions to install the lactyl moiety at the O-3 position of NAG derivatives and to control enantioselective glycosylation. Recent advances are presented and synthetic routes are described according to the main strategy used: (i) based on the availability of starting materials such as glucosamine derivatives; (ii) based on a particular orthogonal synthesis; and (iii) based on the use of other natural biopolymers as raw materials.

## 1. Introduction

**Figure 1:**
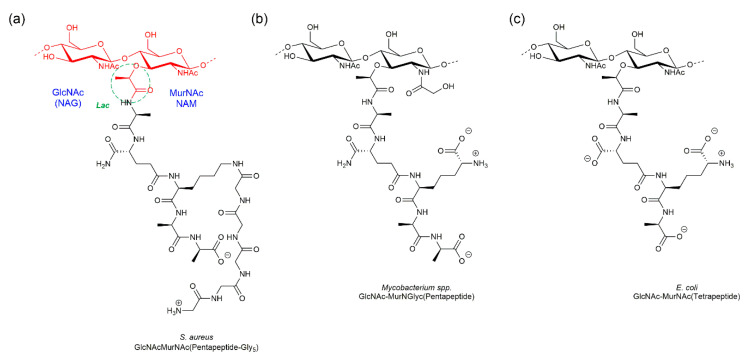
Representation of the monomeric muropeptides frequently found in the peptidoglycan (PGN) of different bacteria. (**a**) the structure of the *Staphylococcus aureus* PGN (a Lys-type PGN) unit is presented. *N*-acetylglucosamine (NAG)–*N*-acetylmuramic (NAM) disaccharide units (red) are linked to each other via NAG-(β-1,4)–NAM linkage. Stem peptides (black), of which the composition is specific for each bacteria species, are attached to the d-lactyl (Lac – highlighted in the green circle) moiety of each NAM. The sequence of the peptide stem attached to the lactyl carboxylate of NAM is l-Ala-d-iGln-l-Lys-d-Ala-d-Ala. (**b**) the structure of a muropeptide of Gram-positive *Mycobacterium* spp., with an L-Ala-D-*i*Gln-*meso*-DAP-d-Ala-d-Ala stem peptide sequence. (**c**) a representative muropeptide of the Gram-negative bacterium *Escherichia coli*.

Peptidoglycan (PGN) is a major component of the cell wall that surrounds most bacteria. It is a macromolecule made of glycan chains, which consist of linear alternating copolymers of β-1,4-linked *N*-acetyl glucosamine (GlcNAc or NAG) and *N*-acetyl muramic acid (MurNAc or NAM) residues. Importantly, these glycan strands carry a lactyl group in the MurNAc moiety that is covalently bound to peptide stems via an amide bond. These stems are used to link glycan strands that are nearby, creating a net-like structure. The composition of the peptide stems is specific for each bacterial species (Figure 1) [1]. The unbranched peptide stems are typically pentapeptides of alternating L- and D-amino acids, usually L-alanine, D-glutamic acid, a diamino acid and two terminal D-alanines. The diamino acid in the third position is usually *meso*-diaminopimelic acid (DAP) in Gram-negative bacteria and l-Lysine is Gram-positive [2]. The diamino acids in the peptide stems allow PGN to “branch out” and connect to neighboring peptide stems, cross-linking adjacent glycan chains [2].

Bacterial PGN, a telltale molecule recognized by the innate immune system from different organisms, has important functions for the survival of bacteria. The structure of the PGN macromolecule determines the shape of these microorganisms; anchors different proteins, which are required for bacteria to interact with the surrounding environment and for the maintenance of envelope integrity; and provides the required mechanical strength to resist osmotic pressure [2]. Importantly, modification of the bacterial process of PGN synthesis, or of PGN composition, can be associated with an altered expression of resistance to different antibiotics. Examples of this effect include resistance to beta-lactams in methicillin-resistant *Staphylococcus aureus* (MRSA) strains, which is dependent on expression of different PGN synthetic enzymes [3], as well as resistance to vancomycin in isolates of *Enterococcus* species, which requires modification of the PGN precursors to reduce the affinity of the antibiotic to its target [4].

PGN metabolism requires (i) the synthesis of PGN building blocks inside bacteria (also known as muropeptide precursors), (ii) their transport across the bacterial membrane, (iii) the polymerization of the PGN scaffold, and (iv) the trimming or degradation of the PGN molecule. These processes take place during the bacterial cell cycle and ensure that PGN, which is a dynamic structure, is remodeled during bacterial growth.

Antibiotics that block PGN synthesis or that lead to changes in the composition of the bacterial cell surface may weaken the cell wall (for example by decreasing the number of crosslinking nodes between different glycan chains and reducing the robustness of the macromolecule to environment insults), lead to alterations of the bacterial shape, and, ultimately, cause bacterial death. Loss of viability may also result from cell lysis, due to the promoted activity of PGN hydrolases [5,6] or due to futile cycling of PGN synthesis and degradation, which depletes cellular resources [7]. PGN hydrolases, or ‘‘lysins’’, are capable of degrading the bacterial cell wall [8]. Lysins can (i) act on the sugar moiety, releasing either glycan fragments with NAG at the reducing end (endo-β-*N*-acetylglucosaminidases) or glycan fragments with NAM at the reducing end (*N*-acetylmuramidases, also known as lysozymes); (ii) act on the peptide moiety (endopeptidases or carboxypeptidases); (iii) hydrolyze the amide bond linking the lactyl group of NAM residues to the peptide stem (N-acetylmuramoyl-l-alanine amidases) [6,7,8]. 

Despite advances in the understanding of PGN biosynthesis and its inhibition by antibiotics, the study of the relevance of PGN structure in bacterial metabolism and of its role in host disease has been hampered by the lack of pure PGN fragments [9].

Chemical synthesis of NAG containing oligosaccharides [10,11,12,13], such as the NAG-NAM disaccharide, is a challenging task due to well-known limitations associated with the enantioselective glycosylation of NAG moieties: (i) the presence of a β-1,4 glycosidic bond requires a multistep synthetic sequence to obtain a regio- and stereoselective assembly of glycosidic bonds, crucial for biological activity; (ii) the use of 2-acetamido-2-deoxyglycosyl donors, due to the formation of 1,2-*O*,*N*-oxazoline intermediates [14,15,16,17], and the use of corresponding acceptors that are poor nucleophiles [18].

As mentioned above, the most demanding aspect in the synthesis of fragments of peptidoglycan is the control of the stereoselectivity of the glycosidic bond. The pursuit of straightforward and stereoselective methods for the assembly of PGN fragments remains an emerging research area. In this review, after an overview of the biosynthesis of bacterial PGN, we highlight recent advances in the synthesis of PGN fragments, focusing on the different strategies, including the selection of donors and acceptors, reaction conditions, stereoselectivity of the glycosylation reaction, and peptide coupling. Recently, chemoenzymatic approaches have been reported to solve problems associated with the orthogonal and multistep processes usually required for PGN synthesis, which will also be covered in this review.

## 2. Biosynthesis of the Bacterial Peptidoglycan

**Figure 2:**
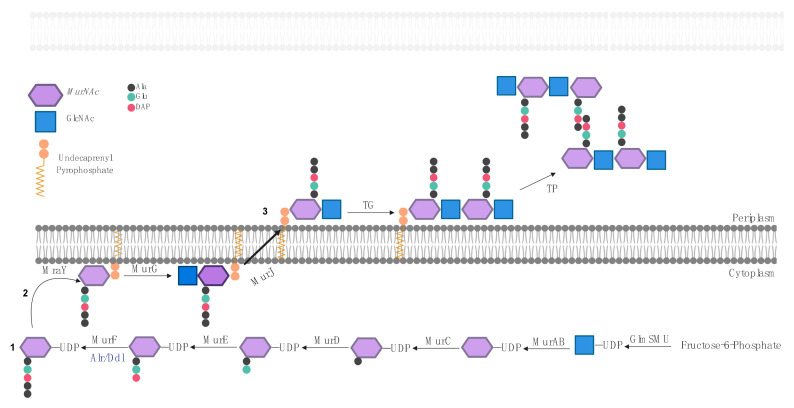
Schematic representation of PGN biosynthesis in Gram-negative bacteria: (**1**) synthesis of a PG precursor in the cytoplasm; (**2**) synthesis of lipid-linked precursors at the inner leaflet of the cytoplasmatic membrane; (**3**) flipping to the outer leaflet of the cytoplasmatic membrane and incorporation of the precursor into nascent PGN by transglycosylation (TG) and transpeptidation (TP) reactions. Enzymes shown in blue synthesize substrates added on the depicted reaction: Alr racemases convert l-Ala to d-Ala and Ddl ligases form the d-Ala-d-Ala dipeptide.

PGN biosynthesis is conserved among Gram-positive and Gram-negative bacteria and occurs in three different locations: (i) synthesis of uridine diphosphate (UDP)-activated PGN precursors in the cytoplasm; (ii) synthesis of lipid-linked precursors at the inner leaflet of the cytoplasmatic membrane; (iii) flipping to the outer leaflet of the cytoplasmatic membrane and incorporation of the precursor into nascent PGN (Figure 2).

PGN biosynthesis starts with the conversion of fructose-6-phosphate into uridine diphosphate-GlcNAc (UDP-GlcNAc) by GlmSMU enzymes [19,20,21,22,23,24]. UDP-GlcNAc is converted to uridine diphosphate-MurNAc (UDP-MurNAc) by MurAB enzymes [25,26,27]. The d-amino acids present in the PG are produced by the MurI and Alr racemases, which convert l-Glu to d-Glu and L-Ala to d*-*Ala, respectively [28,29,30]. The amino acids L-Ala, D-Glu, and L-Lys or DAP are added sequentially by the MurCDE enzymes, respectively, to the lactyl group of UDP-MurNAc [31,32,33,34]. The d-Ala-d-Ala dipeptide present at the carboxyl end of the PGN stem peptide is first formed by Ddl ligases [35] and then incorporated into the UDP-MurNAc-tripeptide by MurF [36,37], forming a UDP-MurNAc-pentapeptide known as the Park nucleotide.

The Park nucleotide is attached to undecaprenyl phosphate in the inner leaflet of the cytoplasmatic membrane by MraY with the release of UDP. The resulting MurNAc-(pentapeptide)-pyrophosphoryl-undecaprenol is known as lipid I [38,39,40]. MurG converts lipid I into lipid II (GlcNAc-*β*-(1,4)-MurNAc-(pentapeptide)-pyrophosphoryl-undecaprenol) by adding the GlcNAc of a UDP-GlcNAc to lipid I with the release of UDP [41,42,43].

The resulting product, lipid II, is flipped from the inner leaflet to the outer leaflet of the cytoplasmatic membrane. There is a debate regarding which enzyme is responsible for this step. Although FtsW and MurJ are the two strongest candidates, the literature favors MurJ as the bona fide flippase [44,45,46,47,48]. More recently, it has been shown that certain enzymes in particular organisms may also exhibit lipid II flippase activity [49].

Precursor incorporation into nascent PG is achieved by reactions of transglycosylation (TG), responsible for extending glycan chains, and reactions of transpeptidation (TP), responsible for connecting adjacent glycan chains through their stem peptides. TG connects the lipid II GlcNac C4 to the MurNAc C1 of the nascent PGN with the release of undecaprenyl pyrophosphate. After the incorporation of the PGN precursor into the PGN macromolecule, undecaprenyl pyrophosphate is subsequentially dephosphorylated, prior to its use in another cycle of glycan synthesis, leading to the removal of one phosphate group, and is recycled for another cycle of Lipid precursor synthesis [50,51].

## 3. Synthetic Approaches towards PGN Fragments from Glucosamine I

Currently, there is a pressing need for new approaches toward the chemical synthesis of PGN fragments. This will permit in vitro studies of the biochemical activity of PBP enzymes [52], which are important targets for the development of new antibiotics. It will also ensure that samples of PGN, a telltale molecule that flags an infected host to the presence of bacteria, are free of contaminants of bacterial origin [53,54].

However, attempts to fully synthesize the PGN molecule in the laboratory in a way that is independent of the use of components (substrates or enzymes) of bacterial origin have faced several restrictions.

The synthesis of NAG-containing oligosaccharides, such as the NAG-NAM disaccharide present in the PGN molecule, poses considerable limitations associated with the enantioselective glycosylation of NAG moieties with respect to both donor and acceptor units. Indeed, the glycosylation of complex aglycones with glycosyl donors bearing 2-acetamido-2-deoxy functionality is usually impractical due to the formation of a 1,2-*O*,*N*-oxazoline intermediate during glycosylation. This is a stable intermediate, which drastically decreases the rate and yield of the glycosylation step, especially when the chosen glycosyl acceptors have poor nucleophilicity or are sterically hindered. Furthermore, the use of glycosyl acceptors bearing 2-acetamido-2-deoxy functionality is considered not to be a good strategy as the amide group in the acceptor molecule can establish a hydrogen bond with the hydroxy group O-4, which decreases nucleophilicity at this position. In order to avoid the formation of the stable 1,2-*O*,*N* oxazoline intermediate, and improve the formation of 1,2-*trans*-glycoside, different authors have used various N-protecting groups in their attempt to synthesize PGN fragments. The N-protecting groups that have been developed differ in their ability to direct the stereochemical outcome of the glycosylation reactions and in their compatibility with diverse carbohydrate synthetic methods.

**Scheme 1:**
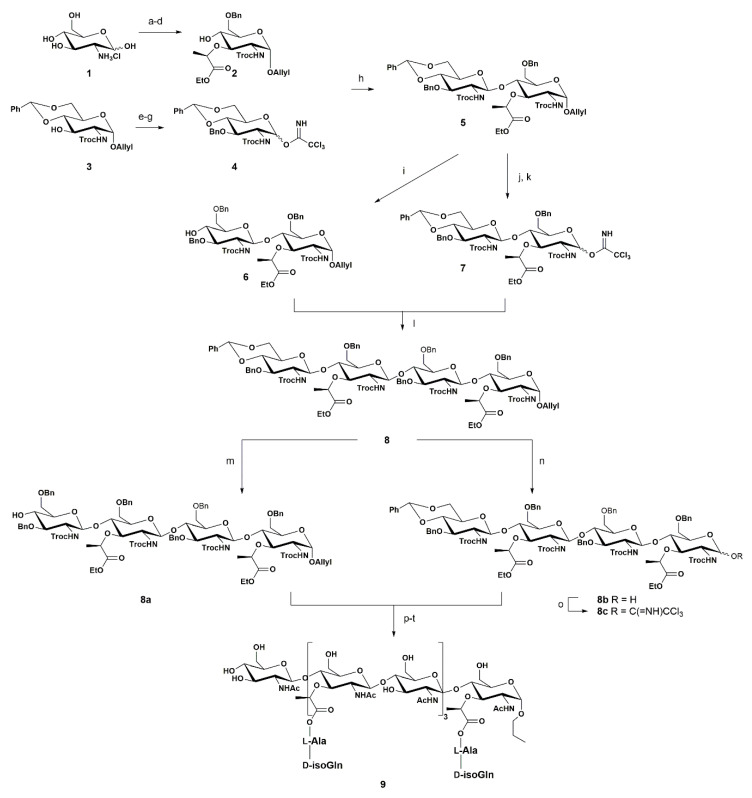
Synthesis of PGN fragment **9** reported by Fukase [55]. (**a**) (i) AllocCl, NaHCO_3_, H_2_O; (ii) AllylOH, DOWEX 50W X8 200–400 mesh, 80 °C; (iii) PhCH(OMe)_2_, *p*-TsOH, 57%; (**b**) NaH then trifluoromethanesulfonyl-l-(*S*)-2-propionic acid benzyl ester, 98%; (**c**) Pd(PPh_3_)_4_, AcOH, DCM, then TrocCl, 58%; (**d**) Me_3_N·BH_3_, BF_3_·Et_2_O, ACN, 87%; (**e**) BnBr, AgO, DCM, 87%; (**f**) Ir complex, H_2_, THF, then I_2_, H_2_O, 99%; (**g**) CCl_3_CN, Cs_2_CO_3_, DCM, quantitative; (**h**) TMSOTf (0.1 equiv.), MS 4 Å, DCM, −15 °C, 88%; (**i**) Me_3_N·BH_3_, BF_3_·Et_2_O, ACN, 52%; (**j**) Ir complex, H_2_, THF, then I_2_, H_2_O, 91%; (**k**) CCl_3_CN, Cs2CO3, DCM, 85%; (**l**) TMSOTf (0.1 equiv.), MS 4 Å, DCM, −15 °C, 79%; (**m**) Me_3_N·BH_3_, BF_3_·Et_2_O, ACN, 60%; (**n**) Ir complex, H_2_, THF, then I_2_, H_2_O, 81%; (**o**) CCl_3_CN, Cs_2_CO_3_, DCM, 83%; (**p**) TMSOTf (0.1 equiv.), MS 4 Å, DCM, −15 °C, 70%; (**o**) Zn–Cu, AcOH, then Ac_2_O, Py, 82%; (**r**) 1 M NaOMe, THF, H_2_O: LiOH, dioxane, THF, H_2_O, 97%; (**s**) HCl·H-l-Ala-d-Glu(OBn)-NH_2_, WSCI·HCl, HOBt, TEA, DCM, 31%; (t) Pd(OH)_2_, AcOH, H_2_ (10 atm), 48%.

In 2001, Fukase and co-workers reported the synthesis of PGN fragments [55], in a follow-up of their previous work [56]. The authors developed an orthogonal synthesis that was able to create the building blocks required to achieve a PGN fragment—the complex octasaccharide (NAG-NAM)_4_ with two amino acid strands linked to every NAM unit **9** (Scheme 1).

The first step in the preparation of acceptor **2** consisted in the protection of the amine group of d-glucosamine with the allyloxycarbonyl (Alloc) group by treatment of glucosamine with AllocCl in the presence of NaHCO_3_ as a base. Next, the anomeric position was protected with the *O*-Allyl group, by reaction with allyl alcohol under acidic conditions using DOWEX as a proton exchange resin at high temperature. The O-4 and O-6 positions were regioselectively protected via acetylidene formation by treatment with benzaldehyde dimethyl acetal catalyzed by *p*-TsOH in 57% yield. This strategy is a well-known procedure for the regioselective protection of both the O-6 and O-4 positions.

For the introduction of the lactyl moiety at the O-3 position, the authors used trifluoromethanesulfonyl-l-(*S*)-2-propionic acid benzyl ester after hydroxy group deprotonation with NaH, and the product was isolated in 98% yield. The N-protection achieved with the introduction of the Alloc group is crucial to install the lactyl moiety at the O-3 position. However, the presence of this group will not permit the correct β-1,4 selectivity during the glycosylation reaction [14]. In order to obtain β-1,4 linkage, the authors replaced the Alloc group with the trichloroethoxycarbonyl (Troc) group. The Alloc group was first removed via treatment with a complex of Pd(PPh_3_)_4_ assisted by AcOH. The palladium complex coordinates with the double bond from the Alloc group, followed by hydrolysis with AcOH. Then TrocCl was added to provide the N-Troc product in 58% yield. The use of Troc as an N-protecting group at the acceptor moiety allowed for the simplified removal of the protecting groups in a later stage of the synthesis. Consequently, selective ring opening was performed to make the O-4 position free in preparation for the glycosylation reaction, using Me_3_N·BH_3_ to reduce the benzylic position, leaving the O-4 position free and providing the acceptor **2** in 87% yield.

The donor **4** was prepared via intermediate **3**, which was obtained from d-glucosamine using a similar approach as that described for the acceptor, consisting of: N-protection with TrocCl, protection of the anomeric position with the *O*-Allyl group, and formation of the acetylidene. To prepare the donor unit, the authors protected the O-3 position with the Bn group, using BnBr, through a S_N_2 reaction catalyzed by AgO and obtained the product in 87% yield. The next step consisted in the conversion of the *O*-Allyl group into a trichloroacetimidate group. Thus, the authors removed the *O*-Allyl group with an Ir complex to isomerize the double bond, promoting the H_2_O nucleophilic attack at the anomeric position. The next step consisted in the treatment of the anomeric position of the donor with CCl_3_CN and Cs_2_CO_3_ as a base, leading to the product **4** in a quantitative yield.

The next step consisted in the glycosylation reaction using **2** as an acceptor and **4** as a donor. Since the trichloroacetimidate group is an excellent leaving group, TMSOTf was used in a catalytic amount, along with molecular sieves, to promote the glycosylation reaction, providing **5** in 88% yield.

Once the disaccharide **5** was obtained, the same rationale was used to produce the tetrasaccharide **8**, including the selective ring opening to provide the acceptor **6**, and replacement of the O-Allyl group with the trichloroacetimidate group to provide the donor **7**. The synthesis of the octasaccharide followed a similar approach via preparation of both acceptor and donor tetrasaccharides **8a** and **8c**, respectively. After obtaining the octasaccharide, the authors attached the dipeptide via solid-phase peptide synthesis. The remaining benzyl groups were removed by a hydrogenation reaction.

Overall, the authors carried out 25 steps to achieve compound **9**. This study [55] was a milestone in the synthesis of PGN fragments. To perform this synthetic plan, the authors had to carefully choose all the protecting groups, in particular the N-protecting group and the protection of the anomeric position. As the *N*-Alloc group, a well-known group that allows many chemical modifications, does not favor β-1,4 glycosidic bond formation in glycosylation reactions, the authors installed the Troc group which is a β-1,4 director.

**Scheme 2:**
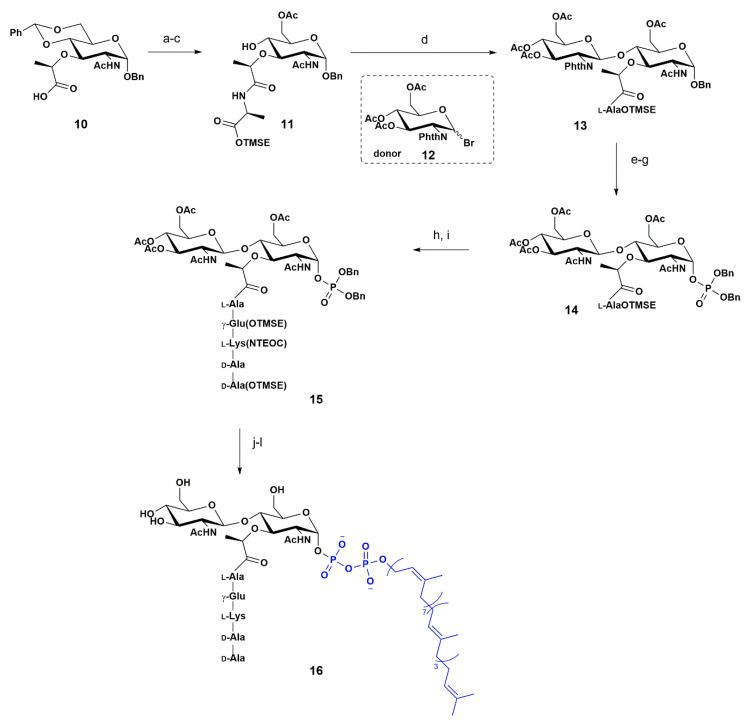
Synthesis of the Lipid II unit **16** (undecaprenyl phosphate, highlighted in blue) reported by Wang [57]. (**a**) HAlaOTMSE, DIEA, HOBt, PyBOP, 1:1 THF:DCM, r.t. (room temperature), 2 h, 81%. (**b**) Catalyst TsOH, MeOH, 75 °C, 30 min, 89%. (**c**) Pyridine, 1 equiv. of AcCl, −30 °C, 15 min, then warm to r.t., 80%. (**d**) AgOTf (3 equiv.), donor (2 equiv.), MS 4 Å, DCM, −40 °C, drybox, 3 h, then overnight, 60%. (**e**) Amino-modified resin, MS 4 Å, butanol, 85 °C, 24 h, then pyridine, Ac_2_O, r.t., overnight, 53%. (**f**) 10%Pd/C, H_2_, MeOH, 1 h, 93%. (**g**) Tetrazole, (BnO)_2_PN(i-Pr)_2_, DCM, −30 °C to r.t., 1 h, then *m*-CPBA, −40 °C to r.t., 1 h, 55%. (**h**) TBAF, THF, r.t., 45 min, 87%. (**i**) H-γ-d-Glu(OTMSE)-Lys(TEOC)-d-Ala-d-AlaOTMSE, DIEA, HOBt, PyBOP, 1:1 THF:DCM, r.t., 2 h, 61%. (**j**) 10%Pd/C, H_2_, MeOH, 1 h, 100%. (**k**) CDI, dry DMF, r.t., 4 h, then MeOH, then undecaprenyl phosphate, r.t., 48 h, 39%. (**l**) TBAF, DMF, r.t., 24 h, then 3% NaOMe, MeOH, 0 °C, 1 h, 35%.

In 2001, Wang and co-workers reported the orthogonal synthesis of the Lipid II unit **16**, Scheme 2 [57]. The authors aimed to develop an appropriate assay system for the observation of catalytic transformations involving PBP1b, a penicillin binding protein that in *E. coli* is involved in the transglycosylation and transeptidation of PGN precursors, since this area of study had been limited by the available amounts of Lipid II.

The synthesis started with the preparation of the acceptor unit **11** from the commercially available MurNAc derivative **10**. The first step was to install the L-alanine unit to avoid the unwanted cyclization of compound **11**, observed when the lactate was protected as an ester. This reaction was followed by removal of the arylidene group and acetylation of the free 6-hydroxy group, affording the acceptor **11** with a 57% yield after three steps.

As a donor, the authors used the commercially available donor **12**, a glycosyl bromide possessing the *N*-phthalimide group. The glycosylation was carried in the presence of AgOTf and, with the participation of the *N*-phathalimido group in **13** that oriented the attack on the β face, the desired compound **13** was isolated in 60% yield. Next, the phtaloyl group in **13** was removed with concomitant *N*-acetylation using an amino-modified resin and acetic anhydride. Compound **13** was then converted to the desired phosphate **14** via consecutive anomeric deprotection and phosphorylation. Thus, hydrogenation of the benzyl group in **13**, using the classical conditions 10% Pd/C afforded the free OH. Phosphorylation was performed in a one-pot, two-step procedure, consisting of the initial formation of the phosphite, followed by in situ oxidation with m-CPBA. The authors used (BnO)_2_PN(*i*-Pr)_2_, followed by *m*-CPBA oxidation, to provide **14** with 27% yield after three steps. Next, the silyl group from alanine was removed by means of treatment with TBAF, followed by pentapeptide coupling using the standard conditions reported by the authors. The final steps relied on the undecaprenyl unit coupling and removal of the protecting groups. The benzyl groups from **14** were removed by hydrogenation, followed by coupling with undecaprenyl phosphate using CDI as a coupling agent to obtain **15**. After removal of the silyl group using TBAF and removal of the acetyl groups under Zemplén’s conditions the Lipid II unit **16** was obtained, consisting of the first synthesis with 12 steps. 

**Scheme 3:**
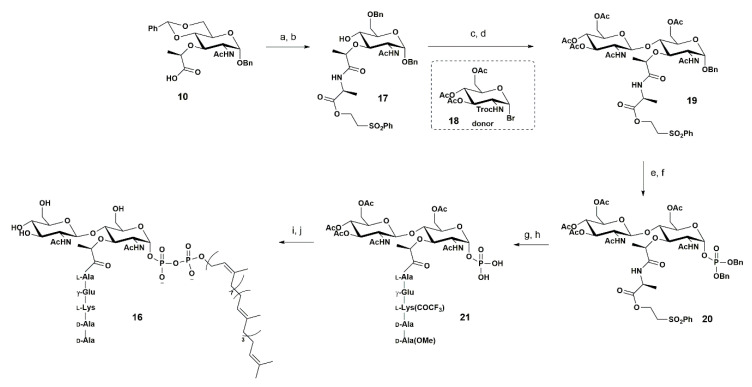
Synthesis of the Lipid II unit **16** reported by Blaszczak [58,59]. (**a**) *N*-methylmorpholine, 2-chloro-4,6-dimethoxy-1,3,5-triazine, DCM, 2 h, 95%; (**b**) Et_3_SiH (3 equiv.), TFA (6 equiv.), DCM, 0 °C, 5 h, 61%; (**c**) AgOTf (3 equiv.), donor (3 equiv.), MS 4 Å (5.5 wt equiv.), DCM, 25 °C, 20 h 84%; (**d**) (1) anhydrous ZnCl_2_,Ac_2_O/AcOH (2:1), 25 °C, 20 h; (2) Zn dust (20 reducing equiv.), 25 °C, 4 h, 67%. (**e**) H_2_, Pd/C, MeOH/THF, 94% yield; (**f**) (1) dibenzyl-*N,N*-diethylphosphoramidite, 1H-tetrazole, DCM; (2) 30% H_2_O_2_, THF, −78 °C to room temperature, 78% yield; (**g**) (1) DBU, DCM; (2) EDCI, NHS, DMF, then peptide and iPr_2_NEt, 46% yield; (**h**) H_2_, Pd/C, MeOH, then pyridine, 91% yield; (**i**) (1) CDI/DMF/THF; (2) undecaprenyl mono-phosphate (bis-amonium salt); (**j**) NaOH/H_2_O/1,4-dioxane, 24% overall yield from **10**.

Blaszczak and co-workers reported, in 2001, the synthesis of the compound **20** [58] and then in 2002 reported the final steps to afford the Lipid II unit **16** (Scheme 3) [59].

The synthesis of **16** started with the coupling of alanine to the commercially available monosaccharide **10** in 95% yield, followed by the reductive opening of arylidene using Et_3_SiH, giving the acceptor **17** in 61% yield. The glycosylation reaction with of **17** with the donor glycosyl bromide **18**, containing the NHTroc group, was carried out in the presence of AgOTf as a catalyst, in 84% yield. Next, the *O*-6 benzyl group was removed, followed by acetylation of the positions O6 and N2 using ZnCl_2_ and Ac_2_O. The N-Troc group was removed using Zn dust in a multistep reaction, yielding **19** in 67% yield. The authors hydrogenated the OBn group, followed by insertion of the phophoryl group, affording **20** in 73% after two steps. The tetrapeptide was coupled using EDCI, followed by hydrogenation of the Bn groups, affording **21** in 41% yield (two steps). The final steps consisted in the coupling of the undecaprenyl moiety using undecaprenyl phosphate and CDI, followed by the removal of the acetyl groups under Zemplén’s conditions, to afford Lipid II unit **16**. This synthesis was carried out in 10 steps.

**Scheme 4:**
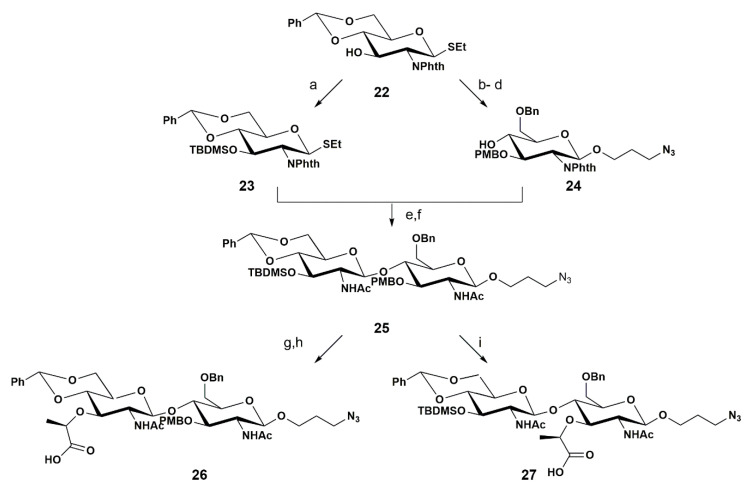
Synthesis of the disaccharides **26** and **27**—precursors of NAM-NAG and NAG-NAM reported by Boons [60]. (**a**) TBDMSOTf, 2,6-lutidine, DCM, 96%; (**b**) PMBCl, NaH, TBAI, THF, reflux, 84%; (**c**) Me_3_N·BH_3_, AlCl_3_, THF, 72%; (**d**) HO(CH_2_)_3_N_3_, NIS, TMSOTf, 67%; (**e**) NIS, TMSOTf, 62%; (**f**) H_2_N(CH_2_)_2_NH_2_, EtOH then Ac_2_O, MeOH, 70%; (**g**) Bu_4_NF, THF, 75%; (**h**) NaH, (*S*)-2-chloropropionic acid, dioxane, **14** (76%) and **15** (66%); (**i**) DDQ, DCM, H_2_O, 79%.

Other groups of researchers have reported alternative strategies to produce molecules that could be used in the synthesis of PGN. In 2002, the Boons group developed the synthesis of a high value disaccharide which can be converted into the intermediates **26** and **27**, precursors of the NAM-NAG and NAG-NAM disaccharides, respectively (Scheme 4) [60].

In their report, the authors started the synthesis of PGN fragments with compound **22,** which possesses the phthalimide (Phth) group as an N-protecting group, a 4,6-*O* arylidene ring, and an SEt group at the anomeric position. The O-3 position of **22** was protected with TBDMSOTf, affording **23** as the donor unit in 96% yield.

Concerning the preparation of acceptor **24**, the synthesis started with the protection of the O-3 position of **22** with the PMB group, using PMBCl and NaH in 84% yield. The next step consisted in the opening of the arylidene ring to make the O-4 position free. This was achieved using Me_3_N·BH_3_ as a reducing agent, and the desired product was obtained in 72% yield. Next, the authors replaced the SEt group with 3-azido-propanol. Subsequent treatment with NIs oxidized SEt to S(O)Et, and this intermediate was attacked by 3-azido-propanol to afford **24** in 67% yield. Interestingly, the authors highlighted that due to the low reactivity of O-4, self-condensation was not observed.

Having both acceptor **24** and donor **23** in hand, the next step consisted in the glycosylation reaction. The activation of the anomeric group with NIS promoted the nucleophilic attack of the hydroxyl group of the acceptor **24** to the donor **23**, affording the corresponding disaccharide in 62% yield. Once the synthesis of the disaccharide was obtained, the N-Phth group was removed by treatment with hydrazine at reflux, followed by an acetylation step, giving the N-acetylated product **25** in 70% yield.

The intermediate **25** was used to prepare both the NAM-NAG precursor **26** and the NAG-NAM precursor **27**. To produce **26** the authors removed the TBDMS group using tetrabutylamoniun fluoride (TBAF) in 75% yield, followed by the introduction of the lactyl moiety. The authors used (*S*)-2-chloropropionic acid after deprotonation of O-3 with NaH, affording the corresponding product in 76% yield. To obtain **27** the authors removed the PMB group using DDQ in 79% yield, followed by the introduction of the lactyl moiety, using the same procedure used for **26**, in 66% yield.

In their synthetic strategy, the authors selected the Phth group as the N-protecting group, since it blocks the nitrogen atom and is only removed by reduction with hydrazine. In addition, a thioglycoside was used as a glycosyl donor due to its versatility and stability and for being a good leaving group when activated with NIS.

Overall, the authors synthesized two disaccharides, which are similar to components of bacterial PGN, in eight steps. Their strategy consisted in first synthesizing the disaccharide unit and afterwards performing the insertion of the lactate moiety. The lactonization of muramic acid derivatives possessing a free O-4 position is a major obstacle. As mentioned above, Blaszczak and co-workers [58,59] addressed this problem by installing the l-alanine in the glycosyl acceptor for the synthesis of a disaccharide. The route developed by the Boons group addressed this challenge by synthesizing a disaccharide and thereafter incorporating the lactate unit; consequently, one disaccharide can be used for the synthesis of both PGN units (MurNAc-GlcNAc and GlcNAc-MurNAc).

The N-Troc group of the glycosyl donor is known to be very reactive [18], directing glycosylation exclusively to produce 1,2-*trans* linked glycosides via neighboring group participation. The authors found that glycosyl donors that were protected with phthalimido groups led to high-yielding glycosylations. This N-protecting group is compatible with strong basic conditions used for the incorporation of the lactate moiety, whereas the Troc group would not survive these conditions.

**Scheme 5:**
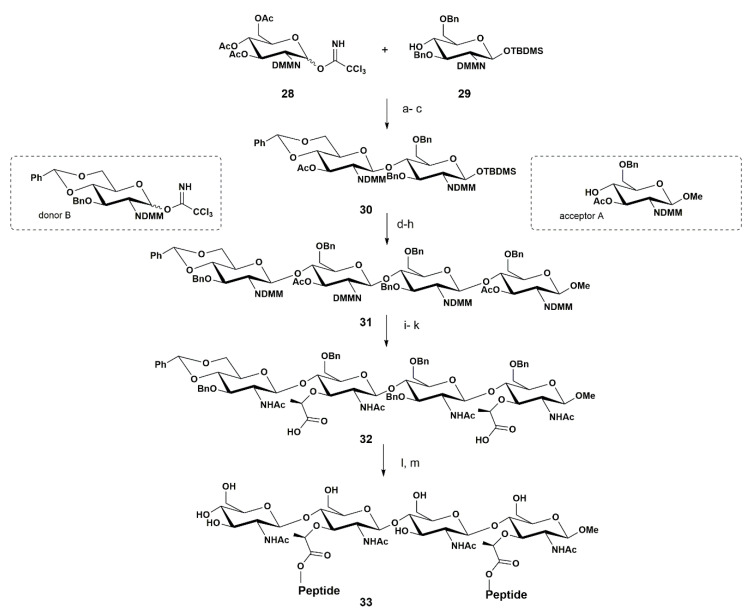
Synthesis of the tetrasaccharide with pentapeptides attached to NAM units, **33**, reported by Mobashery [61]. (**a**) TfOH, 71%. (**b**) (1) NaOMe, Amberlite IR-120 (H+), (2) benzaldehyde dimethylacetal, *p*-TsOH, 60% in two steps; (**c**) Ac_2_O, 85%; (**d**) *n*-Bu_4_NF, 75%; (**e**) Cl_3_CCN, DBU; (**f**) acceptor A, TfOH, 55% in two steps; (**g**) BH_3_.NMe_3_, BF_3_.OEt_2_, 62%; (**h**) donor B, TfOH, 68%; (**i**) (1) NaOH, dioxane/water (4:1); HCl, pH 3.0; (2) Ac_2_O, 48% in two steps; (**j**) NaOMe, 74%; (**k**) NaH, (*S*)-2-chloropropionic acid, 46%; (m) 4-nitrophenyl trifluoroacetate, pyridine; peptide (prepared as CF_3_CO_2_H salt), 41%; (**l**) 60% AcOH; H_2_, Pd/C, 76%.

Soon after, in 2004, Mobashery and co-workers reported the synthesis of another PGN fragment—a tetrasaccharide, with two pentapeptides attached to the NAM units, **33** (Scheme 5) [61].

These authors started their synthesis by preparing both **28** and **29** from glucosamine via orthogonal synthesis. This began with protecting the amino group of glucosamine with the dimethyl maleimide (DMM) group, using 2,3-dimethylmaleic anhydride followed by acetylation of the hydroxy groups, affording the corresponding product in 55% yield after two steps. This *N*-DMM glucosamine intermediate was the precursor for both the donor **28** and the acceptor **29**.

To prepare **28** the authors selectively deprotected the anomeric acetyl group using hydrazine acetate to give a free OH group at the anomeric position in 68% yield. Then, at this position, the trichloroacetimidate group was introduced by treatment with CCl_3_CN and DBU as a base, providing the donor **28** in 85% yield.

Concerning the preparation of acceptor **29**, the authors selectively removed the anomeric acetyl group from peracetylated N-DMM glucosamine, using hydrazine acetate (68% yield), followed by silylation of the anomeric hydroxyl group using TBDMSCl and imidazole to afford the silylated product in 78% yield. Next, the remaining acetyl groups were removed using NaOMe in methanol and the product was obtained in 73% yield. The benzylation of the O-3 and O-6 was performed using Bu_2_SnO and BnBr, and the acceptor **29** was isolated in 73% yield.

Having both donor **28** and acceptor **29** in hand, the glycosylation reaction was performed. This step was carried using TfOH as a promotor, producing the desired disaccharide in 75% yield. Three additional steps were needed to produce disaccharide **30**. In the first step there was the removal of the acetyl group using NaOMe that was quenched by the use of a proton exchange resin (Amberlite IR-120). In the second step, the authors executed the subsequent reaction with benzaldehyde dimethylacetal, leading to the formation of 4,6-*O* arylidene, and then O-6 and O-3 benzylation, (60%, in the two steps). The last step consisted in the acetylation of the remaining free O-3 position with acetic anhydride, affording **30** in 85% yield.

Glycan chain elongation using **30** as a substrate was then required. In order to achieve this, the TBDMS group was removed with TBAF in 75% yield. The authors observed that for long reaction times, the yield decreased due to hydrolysis of the acetyl group. Then, the trichloroacetimidate group was introduced, followed by a glycosylation reaction with an acceptor A (Scheme 5) possessing both O-6 benzylated and O-3 acetylated and an OMe group at the anomeric position, affording the corresponding trisaccharide in 55%. Next, the selective opening of the 4,6-*O*-arylidene ring with Me_3_N·BH_3_ gave the corresponding trisaccharide with the free O-4 position in 62% yield.

The next glycosylation reaction was performed using this trisaccharide as an acceptor and the monosaccharide B as a donor (Scheme 5), possessing a 4,6-*O*-arylidene ring, O-3 protected with a benzyl group, and the trichloroacetimidate group at the anomeric position. This glycosylation reaction was once again catalyzed by TfOH, affording **31** in 68%

At this stage the DMM group was no longer needed, and it was removed by treatment of **31** with NaOH in dioxane/water (4:1), quenched with HCl until reaching pH 3, followed by a reaction with acetic anhydride. The corresponding *N*-acetylated product was isolated in 48% yield. To introduce the lactyl moieties, the authors removed the *O*-acetyl groups using NaOMe with 74% yield, followed by treatment with (*S*)-2-chloropropionic acid (after deprotonation with NaH), and **32** was obtained in 46% yield.

The final steps involved the coupling of the tetrasaccharide with a synthesized pentapeptide (prepared as a CF_3_CO_2_H salt) in a 41% yield, and removal of the benzyl groups by hydrogenation, affording **33** in 76% yield.

The authors reported the synthesis of the tetrasaccharide **33** in 18 steps. Their strategy relied on the preparation of a disaccharide moiety possessing two O-3 positions that were differently protected (OAc and OBn), which once glycosylated with a donor and acceptor with the same O-3 protective groups led to a tetrasaccharide, which allowed alternated installations of the lactyl moiety. This was the first report on the synthesis of a tetrasaccharide with the pentapeptide linked to the two *O*-3 lactyl groups. 

Several PGN fragments, mono-, di-, tetra-, and octasaccharides with the pentapeptide assembled were synthesized by the Fukase group in order to understand how the innate immune response was triggered [62]. The synthesis of these PGN fragments relied on a previous report from the same authors [55], using Troc as the protecting group for the amine moiety and the trichloroacetimidate group at the anomeric position. With these studies, the authors determined that the minimal recognition pattern for hPGRP-L, a human protein capable of binding bacterial PGN fragments, was the NAM unit linked to three amino acids. Furthermore, these authors also used the assembled synthetic PGN fragment to discover that the NAM unit linked to two amino acids (MDP) was recognized by NOD2, a protein that is involved in the activation of an innate immune response in murine cells.

**Scheme 6:**
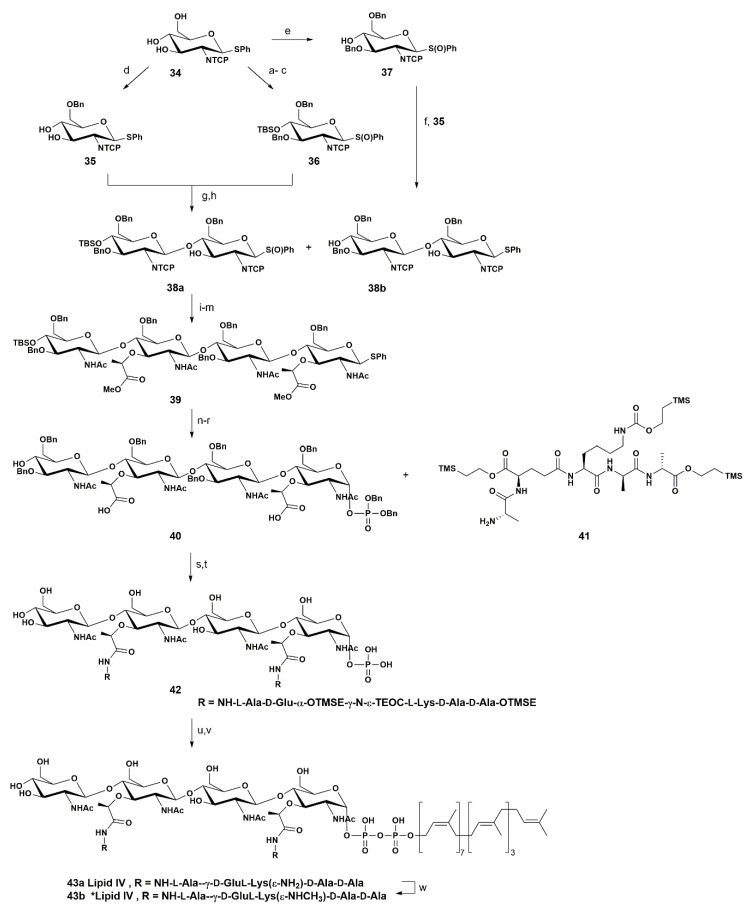
Synthesis of the heptaprenyl—Lipid IV **43b** reported by Walker [63]. (**a**) Bu_2_SnO, toluene, reflux, then Bu_4_NI, BnBr, reflux, 51%; (**b**) TBSOTf, 2,6-lutidine, CH_2_Cl_2_, 91%; (**c**) *m*CPBA, CH_2_Cl_2_, −78 °C to −60 °C, 93%; (**d**) (1) (Bu_3_Sn)_2_O, MeOH, reflux; (2) Bu_4_NI, BnBr, toluene, 91 °C, 75%; (**e**) *m*CPBA, CH_2_Cl_2_, −78 °C to −60 °C, 91%; (**f**) Tf_2_O, DTBMP, ADMB, MS 4Å, CH_2_Cl_2_, −60 °C to −40 °C, 58%; (**g**) Tf_2_O, DTBMP, ADMB, MS 4Å, CH_2_Cl_2_, −60 °C to −30 °C, 75%; (**h**) *m*CPBA, CH_2_Cl_2_, −78 °C to −60 °C, 86%; (**i**) Tf_2_O, DTBMP, ADMB, MS 4Å, CH_2_Cl_2_, −40 °C, 77%; (**j**) (NH_2_CH_2_)_2_, THF/CH_3_CN/EtOH (1:2:1), 60 °C; (**k**) Ac_2_O, MeOH/H_2_O (5:1), r.t., 75% two steps; (**l**) NaH, *S*-(−)-2-bromo-propionic acid, THF, 0 °C to r.t.; (**m**) TMSCHN_2_, benzene/MeOH (3:1), 0 °C, 70% two steps; (**n**) NIS, CH_3_CN/H_2_O (5:1), r.t., 75%; o) 1*H*-tetrazole, (*i*-Pr)_2_NP(OBn)_2_, CH_2_Cl_2_, −40 to −20 °C; (**p**) *m*CPBA, CH_2_Cl_2_, −40 °C to r.t., 84% two steps; (**q**) TBAF, THF, 0 °C to r.t.; (**r**) 1.3 M KOH, THF/H_2_O (10:1), r.t., 64% two steps; (**s**) HATU, DIEA, DMF, r.t., 60%; (**t**) Pd(OH)_2_/C, H_2_, 44%; (**u**) ammonium heptaprenyl phosphate, 1,1′-carbonyl diimidazole, THF, r.t., then **30**, SnCl_2_, DMF, r.t., 50%; (**v**) TBAF, DMF, r.t., 69%; (**w**) (CH_3_[14]CO)_2_O, toluene/16 mM NaOH in MeOH (1:1), sonication, 37 °C, 50%.

In 2007 Walker and co-workers reported the synthesis of a PGN precursor that may be used by bacterial enzymes that are involved in the assembly of the PGN macromolecule—the heptaprenyl-lipid IV **43b**, which consists of a tetrasaccharide (NAG-NAM)_4_ linked to a diphospholipid at the anomeric position (Scheme 6) [63].

The strategy proposed by these authors relied on the preparation of a tetrasaccharide possessing alternated free hydroxyl groups at the O-3 position that could then be used to install the lactyl unit and the posterior attachment of the peptide chain, while the lipid moiety was introduced using a thioglycoside as donor.

The authors started the synthesis with the common starting material **34** that was converted into monosaccharides **35**, **36**, and **37**—building blocks required to assemble the desired tetrasaccharide. Glucosamine derivative **34** was converted into **35** in three steps. In the first step, they used an O-6 and O-3 benzylation using Bu_2_SnO and BnBr in 51% yield. The second step involved O-4 silylation by means of treatment with TBSOTf and lutidine in 91% yield. In the third and final step, the authors performed the oxidation of the thioglycoside, with *m*CPBA affording **35** in 93% yield.

The production of **36** and **37** did not require so many steps. Treatment of **34** with (Bu_3_Sn)_2_O, followed by the addition of BnBr/TBAI (O-6 benzylation) led to **36** in 75% yield. The donor **37** was obtained by oxidation of **24** with *m*CPBA.

Glycosylation of **35** with **36** mediated by Tf_2_O followed by oxidation with *m*CPBA led to formation of the disaccharide **38a** in 86% yield. N-protection with the tetrachlorophthalimide (TCP) group hindered the O-3 glycosylation reaction, which was expected due to the presence of the free hydroxyl group at O-3, and permitted the formation of a β-1,4 glyosidic bond. The disaccharide **38b** was obtained in 58% yield via the inverted addition of **37** to the acceptor **35**.

The glycosylation of the two disaccharides (**38a** and **38b**) afforded the desired tetrasaccharide in 77% yield. Next the N-TCP group was replaced by the N-acetyl group via treatment of the tetrasaccharide with hydrazine and subsequent N-acetylation with acetic anhydride in 75% yield (two steps). The generated tetrasaccharide possessed alternated free O-3 positions, which were ready to receive the lactyl moiety. The authors introduced the lactyl moiety using *S*-(*–*)-2-bromo-propionic acid, followed by an *O*-alkylation step, affording **39** in 70% yield.

In order to install the phosphonate moiety at the anomeric position, which yielded compound **40**, the compound **39** was subjected to a sequence of five synthetic steps, involving the oxidation of SPh with NIS in 75% yield, treatment with (*i*-Pr)_2_NP(OBn)_2_ and subsequent oxidation with *m*CPBA in 84% yield (two steps), TBS group removal, and hydrolysis of the ester groups.

The coupling of the silyl-protected pentapeptide **41**, which had been previously synthesized using Fmoc chemistry, with tetrasaccharide **40** was performed using standard peptide coupling conditions. This approach led to tetrasaccharide **42**, which was obtained after global hydrogenolysis in 44% yield.

Next, the coupling of the heptaprenyl phosphate [64] to the anomeric phosphate present in compound **42** was performed with 1,1′-carbonyl diimidazole in 50% yield. The final steps consisted in the removal of the silyl groups, which resulted in the heptaprenyl—Lipid IV **43a**. In order to perform certain enzymatic assays, the authors further converted **43a** into **43b** by treatment with [14] C-acetic anhydride.

In this report the authors established an orthogonal synthetic plan based on the use of a building block possessing the amino group protected with tetrachlorophthalimide (TCP) and the thiophenol group at the anomeric position. The authors then used the synthesized PGN building blocks, together with a bacterial enzyme, PBP1a, a peptidoglycan glycosyltransferase from *E. coli*, to elongate the PGN strand. They successfully mimicked the natural process and were able to convert the synthetic tetrasaccharide into octasaccharide in 51% yield.

**Scheme 7:**
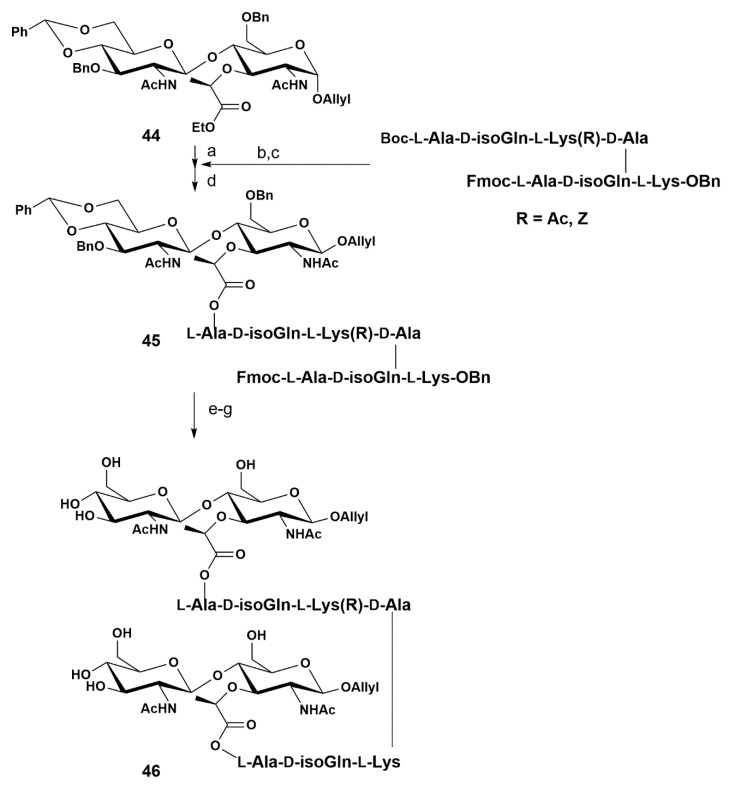
Synthesis of cross-linked PGN fragment **46** reported by Fukase [65]. (**a**) LiOH, THF/1,4-dioxane/H_2_O = 4/2/1, quantitative; (**b**) TFA; (**c**) HCl.Et_2_O; (**d**) Peptide, HATU, Et_3_N, DMF/DMSO (1:1), 75%, three steps; (**e**) 10% TFA/DCM; (**f**) 20% piperidine/DMSO; (**g**) H_2_, Pd(OH)_2_, AcOH.

More recently, Fukase and co-workers reported the first synthesis of cross-linked PGN fragments that are frequently found at the cell wall of *Streptococcus pneumoniae* bacteria [65]. The authors were able to use the disaccharide **44** from previous reports [55,66] as a key intermediate in order to cross-link the peptide chains (Scheme 7).

The ester group from disaccharide **44** was hydrolyzed to the carboxylic acid and the Boc group from the peptide substrate was removed. The peptide was then coupled to the disaccharide using the standard procedure with HATU, affording **45** in 75% yield. The authors further attempted to introduce two glycan chains to the two l-Ala residues of the linked structure. However, the desired compounds were not obtained due to insufficient reactivity of the amino groups.

Next, the benzylidene acetal was removed after treatment with 10% TFA. The authors reported difficulties associated with the removal of the Fmoc group from the peptide chain due to low solubility, but it was accomplished after treatment with 20% piperidine.

The coupling of the second disaccharide moiety was carried out using the same conditions as before, affording the corresponding product in 26–42% yield, depending on the lysine protecting group (Z or Ac). Final hydrogenation removed all the benzyl groups and **46** was afforded in quantitative yield.

The authors investigated the biological activity of all the synthetic PGN fragments and found that the cross-linked PGN fragment is not the major ligand of human NOD2.

This synthetic route developed by Fukase and coworkers established a plan that allows access to longer PGN structures, which might be useful for the investigation in innate immunostimulation of PGN. Indeed, on the basis of this study and PGN fragments, other biological activities can be investigated, as well as the determination of the structural requirements for other types of PGN recognition molecules including peptidoglycan recognition proteins (PGRPs) and PGN-recognizing lectins.

**Scheme 8:**
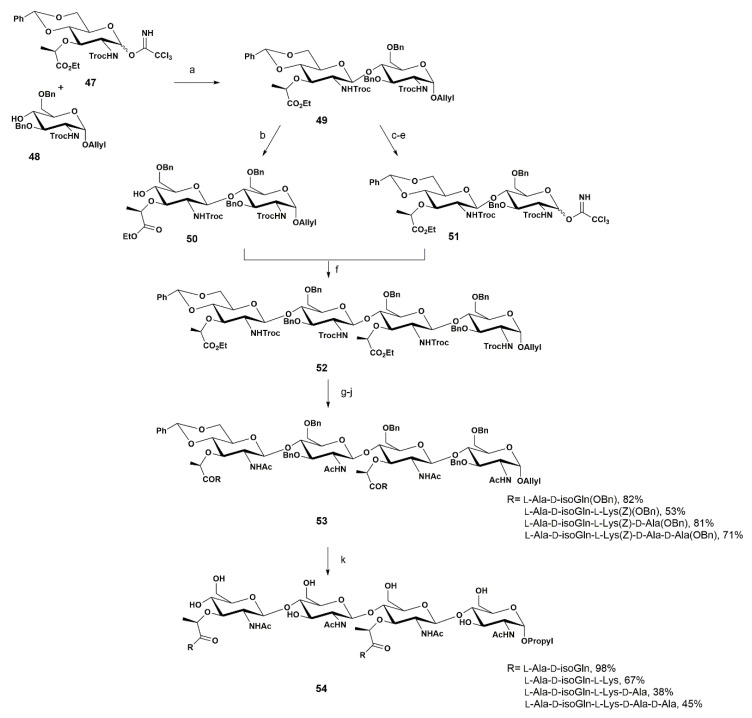
Synthesis of the PGN fragments **54** reported by Fukase [67]. (**a**) TMSOTf, CH_2_Cl_2_, −15 °C, MS 4Å, 20 min, 84%; (**b**) Me_3_N BH_3_/BF_3_·Et_2_O, ACN, 1 h, 83%; (**c**) [Ir(cod)H-(MePh_2_P)_2_]PF_6_, THF, 1.5 h; (d) I_2_/H_2_O, 30 min, 81%; (**e**) CCl_3_CN/Cs_2_CO_3_, DCM, 30 min, quantitative; (**f**) TMSOTf, DCM, MS 4Å, 40 min, 16%; (**g**) Zn/Cu, AcOH,3 h, Ac_2_O/Py, 2 h, 62%; (**h**) LiOH, quantitative. For the condensation: (**i**) WSCD/HOBt/DMF/TEA, HCl·l-Ala-d-isoGln(OBn) or HCl·L-Ala-d-isoGln-l-Lys(Z)(OBn); (**j**) HATU/DMF/TEA, HCl·l-Ala-d-isoGln-l-Lys(Z)-d-Ala(OBn) or HCl·l-Ala-d-isoGln-l-Lys(Z)-d-Ala-d-Ala(OBn); (k) H_2_/Pd(OH)_2_, AcOH.

Some years later, the Fukase group reported the synthesis of several disaccharide and tetrasaccharide fragments, which were used to investigate their ability to stimulate human Nod2 (hNod2)-dependent responses [67]. Nod2 can recognize specific fragments of the bacterial cell-wall PGN (the minimum ligand is the muramyl dipeptide (MDP)). Enzymatic digestion of PGN appears to be important for Nod2 recognition. PGN is degraded by muramidase or glucosamidase, producing two types of glycan sequence—glycans containing NAGβ(1-4)NAM or NAMβ(1-4)NAG. Thus, the authors prepared fragments of diverse composition to understand whether the sequence of these PGN fragments could affect an immune response that was dependent on the innate immune receptor Nod2 (Scheme 8).

In their work, the authors applied previously reported synthetic studies [68,69]. Compound **49** was synthesized in 84% yield via the glycosylation reaction of **47** with **48** mediated by TMSOTf as a catalytic promotor. Disaccharide **49** was converted into the acceptor **50** and donor **51**, to assemble the desired tetrasaccharide **52**. Preparation of acceptor **50** involved the regioselective opening of the arylidene ring to free the O-4 position under standard reductive conditions using Me_3_N·BH_3_ in ACN for 1 h, affording **50** in 83% yield. The donor **51** was prepared in three steps from **49**, involving different reactions: (1) the isomerization of the allyl double bond, using an Ir complex; (2) the subsequent hydrolysis assisted by iodine, in 81% yield for these two steps; and (3) the final installation of the trichloroacetimidate group at the anomeric position in a quantitative yield.

Tetrasaccharide **52** was obtained after glycosylation using TMSOTf as a promotor in 16% yield. In their work, the authors also described the use of glycosyl *N*-phenyltrifluoroacetoamidate as a donor, due to its high reactivity and better stability when compared to the corresponding trichloroacetimidates. The formation of byproducts such as the *N*-glycosyl trifluoroacetamides is suppressed, since the *N*-phenyl group of the eliminated *N*-phenyltrifluoroacetamide prevents the undesirable attack of the amide on the cationic intermediates. The authors also performed the glycosylation using a *N*-phenyltrifluoroacetimidate donor by increasing the equivalent amount of acceptor **50** (donor/acceptor ratio 1:1.5), with an improved yield of 61%, and preserving the selectivity.

Next, the Troc group from tetrasaccharide **52** was removed using a Zn/Cu in AcOH for 3 h, followed by an N-acetylation reaction (acetic anhydride in pyridine for 2 h). The saponification of the ethyl esters with LiOH afforded the corresponding product in a quantitative yield. 

The following steps involved the condensation of the tetrasaccharide with a library of peptides with a composition found in the bacterial PGN (i.e., di-, tri-, tetra-, and pentapeptides). Thus, different protocols were used depending on the peptide chain required. After the final hydrogenation, a library of PGN fragments **54** was obtained in yields ranging from 38% to 98%.

Fukase and co-workers demonstrated that hNod2 recognition is dependent on the glycan sequence. The authors compared the activities of glycans with the same peptide moieties, and showed that (MurNAcβ(1-4)GlcNAc)_2_-containing structures exhibited stronger activity than those containing (GlcNAcβ(1-4)MurNAc)_2_. These results indicate that differences in the enzymatic degradation process may affect the host’s immunomodulation process.

**Scheme 9:**
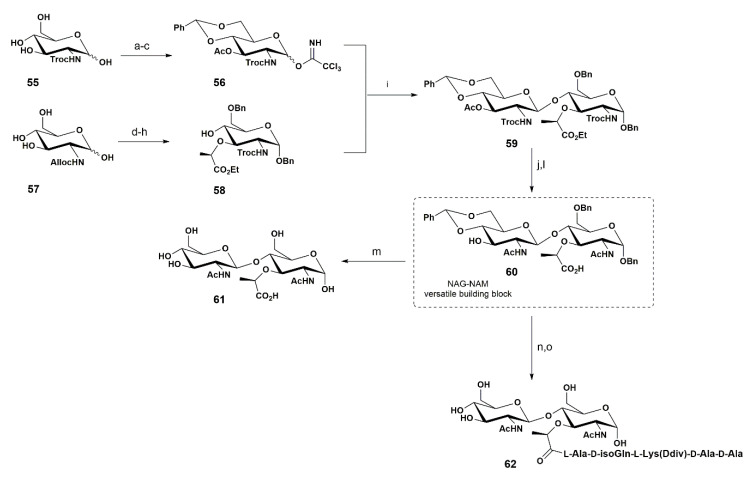
Synthesis of the PGN fragment **62** reported by Marques [70]. (**a**) (1) PhCHO, ZnCl_2_, MS 3Å, r.t., overnight; (2) Ac_2_O, pyridine, r.t., overnight 78%; (**b**) morpholine, EtOAc, r.t., overnight, 87%; (**c**) CCl_3_CN, Cs_2_CO_3_, CH_2_Cl_2_, r.t., 2 h, 74%; (**d**) BnOH, AcCl, 80 °C, 3 h, 61%; (**e**) PhCHO, ZnCl_2_, overnight, 72%; (**f**) ethyl l-(*S*)-2- trifluoromethanesulphonyloxy-propionate, NaH, CH_2_Cl_2_, r.t., 3 h, 68%; (**g**) Pd(PPh_3_)_4_, AcOH, TrocCl, r.t., 82%; (h) BH_3_Me_2_N, BF_3_OEt_2_, r.t., 3 h, 72%; (**i**) TMSOTf, DCM, MS 3 Å, -15 °C, 52%; (**j**) Zn-Cu, THF: AcOH: Ac_2_O (1:1:1), then Ac_2_O/pyridine, 63%; (**l**) LiOH, THF:1,4-dioxane:H_2_O, 69%; (**m**) Pd(OH)_2_, H_2_, AcOH, r.t., quantitative; (**n**) peptide coupling via Fmoc-solid-phase synthesis; (**o**) TFA (1% CH_2_Cl_2_), r.t.

More recently, our group reported the synthesis of a NAGβ-(1-4)-NAM moiety **61**, which is crucial for the preparation of synthetic components of a bacterial peptidoglycan (Scheme 9) [70].

In 2016, Fukase and co-workers reported a chemoenzymatic approach to the large-scale synthesis of a *meso*-DAP type PGN from *Mycobacterium* [71]. Since *meso*-DAP has been reported to be the most potent activator of Nod2, there was a need to develop a large-scale synthesis protocol. The use of l-aminocyclase and d-aminocyclase was a key factor in the production of *meso*-DAP on a large scale in order to proceed with the biological studies.

The proposed route involved the preparation of both donor **56** and acceptor **58** from *N*-Troc glucosamine **55** and *N*-Alloc glucosamine **57**, respectively. The synthetic sequence relied on standard orthogonal protection of the hydroxyl groups, involving four steps for the preparation of **56**: (i) the formation of the O-4,6 arylidene; (ii) an O-3 and O-1 acetylation, via treatment with benzaldehyde dimethyl acetal and ZnCl_2_, the crude dissolved in pyridine and acetic anhydride, in 78% yield (two steps); (iii) the selective removal of the anomeric acetyl group using morpholine, in 87% yield; and (iv) the final establishment of the desired glycosyl trichloroacetamidate using CCl_3_CN/Cs_2_CO_3_, affording **56** in 74% yield, which was used in the glycosylation reaction without further purification.

The acceptor **58** was prepared in five steps, involving the following reactions: (i) the benzylation of the anomeric position using BnOH and AcCl in 61% yield; (ii) the formation of the 4,6-O arylidene, in 72% yield; (iii) the introduction of the lactyl moiety at O-3 using trifluoromethanesulfonyl-l-(*S*)-2-propionic acid ethyl ester, in 68% yield; (iv) the removal of the Alloc group by treatment with Pd(PPh_3_)_4._ followed by in situ N-protection with TrocCl, affording the corresponding product in 82% yield; and (v) a final selective arylidene ring opening using the classical procedure with Me_3_N·BH_3_, affording **58** in 72% yield.

The glycosylation reaction was carried using TMSOTf as a promoter, giving the product **59** with the desired β-1,4 selectivity, in 52% yield. The exchange of both trichloroethyl carbamate groups by the natural N-acetyl group was performed by addition of Zn–Cu in AcOH/Ac_2_O/THF, followed by N-acetylation, in 63% yield. The key intermediate **60** was obtained after hydrolysis with LiOH, which simultaneously led to the removal of the O-3 acetyl group and lactic acid formation, in 69% yield.

Disaccharide **60** constitutes a versatile intermediate, allowing further regioselective manipulation at O-3 and O-4 (after arylidene ring opening) positions, as well as the insertion of a peptide chain at the lactate moiety.

Accordingly, NAG-NAM disaccharide **61** was obtained in quantitative yield by hydrogenation using Pd(OH)_2_ on charcoal (removal of the benzyl groups at the O-1 and O-6 positions of the muramyl residue and the benzylidene ring at the O-6 and O-4 positions of the NAG unit).

Disaccharide **60** was also used to prepare the glycopeptide **62** by coupling with a pentapeptide, previously attained via solid-phase synthesis using HMPB-AM resin, followed by cleavage from the resin upon treatment with trifluoroacetic acid solution.

This work was a follow-up to previous approaches reported by us, such as one-pot regioselective protection and orthogonal approaches towards key intermediates to achieve NAG-NAM moieties [14,72]. In this approach, the conjugation of the different protecting groups allowed for a shorter synthetic sequence and a complete removal of protecting groups in a late stage of the synthesis. In particular, the choice of the anomeric benzyl group facilitated the removal of protecting groups in one step. This work represents a useful route to obtain the NAG-NAM disaccharide moiety, as well as non-natural derivatives.

## 4. Synthetic Approaches towards the Carbohydrate Backbone of PGN from Biopolymers

In the previous section, we described the different approaches developed to address the synthesis of the complex PGN structure from a scaffold of residues of NAG derivatives. The main difficulty in the synthesis of PGN fragments involves stereoselective glycosylation involving NAG derivatives [10,73,74]. Approaches to assembling the complex PGN fragments have been described, and include one-pot regioselective protection and orthogonal approaches to achieve key intermediates as precursors of NAG-NAM moieties [12,14,15,16,17,61,63,65,70,71,75,76].

**Scheme 10:**
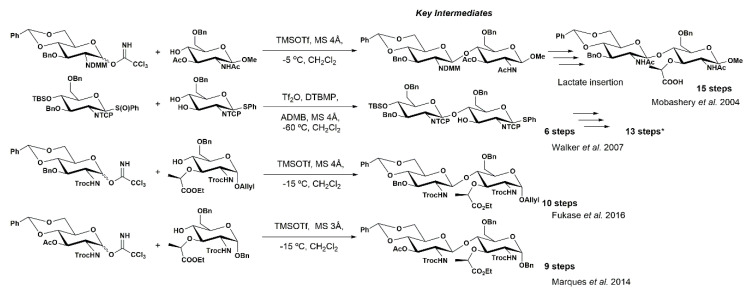
Reported multistep synthesis of NAG-NAM intermediates via orthogonal routes from glucosamine.

We and others have been focused on establishing straightforward routes to achieve PGN fragments and also on protection–deprotection methodologies of NAG-containing saccharides (Scheme 10) [12,61,63,65,70,71,72,75,76]. All these synthetic methods involve a high number of steps required to achieve a key intermediate that possesses the lactyl moiety installed at the O-3 position of the NAG moiety. These long multistep sequences are a puzzling game of compatible protecting groups, due to the difficulties associated with the establishment of the β-1,4 glyosidic bond of NAG-NAM. The success of a particular synthetic sequence relies on the proper choice of protecting groups, in particular the N-protecting group (e.g., Troc, TCP, MDM) [14,77,78], which enable enantioselective control over glycosylation, as well as regioselective insertion of the lactyl moiety at the O-3 position of a modified and alternated (in the case of oligosaccharides) NAG unit.

During recent years, our group embraced the challenge of synthetizing NAG-NAM containing oligosaccharides and its precursors, using as starting material biopolymers, with the β-1,4 glycosidic bond already installed.

In order to explore unprecedented synthetic studies, in order to develop alternatives to the synthesis of PGN fragments, we were inspired by the particular sugar arrangement of PGN (Figure 1) and its resemblance to other biopolymers found in nature, such as chitin and chitosan. Chitin is one of the most abundant biopolymers in the world and consists of β-(1,4)-*N*-acetylglucosamine (NAG), sharing the same basic carbohydrate backbone as that of PGN.

**Scheme 11:**
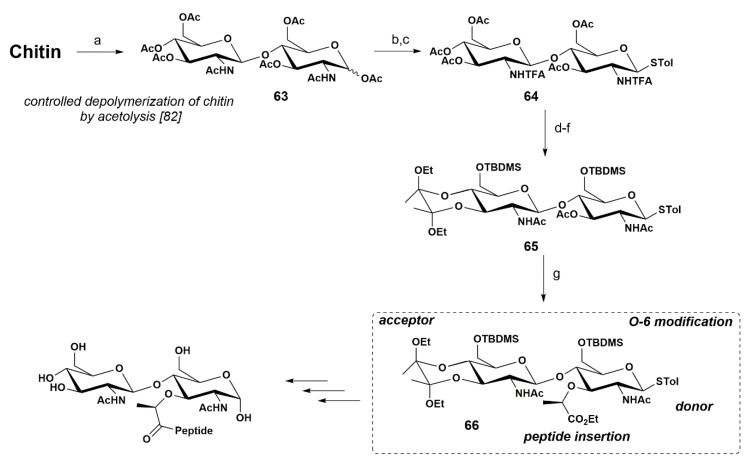
Synthesis of the NAG-NAM precursor **66** from peracetylated chitobiose **63** reported by Marques group [79]. (**a**) Ac_2_O, H_2_SO_4_, 55 °C, 3 h, then r.t., 16 h, and 55 °C, 1 h, 13%; (**b**) TFAA, Pyr, 120 °C, 15 min, 76%; (**c**) TolSH, BF_3_OEt_2_, DCM, r.t., overnight, 80%; (**d**) (1) MeONa, MeOH, r.t., overnight. (2) butane-2,3-dione, *p*-TsOH, triethylorthoformate, EtOH, 60 °C, 16 h, 58%; (**e**) TBDMSCl, DMAP, TEA, CH_2_Cl_2_, 56%; (**f**) (1) NaOH 2 M, THF, 80 °C, 3 h, (2) Ac_2_O, pyridine, CH_2_Cl_2_, r.t., overnight, 56%; (**g**) (1) NaH, DCM, 30 min; (2) Ethyl (*S*)-2-(trifluoromethylsulfonyloxy)propionate, r.t., 16 h, 38%.

In 2018 we established a procedure to achieve an advanced precursor of the NAG-NAM unit via orthogonal synthesis, from peracetylated chitobiose, obtained after chitin chemical hydrolysis, as shown in Scheme 11 [79].

Peracetylated chitobiose **63** was obtained on a gram scale from the chemical and controlled depolimerization of chitin via acetolysis [80,81], using an improved procedure from that originally described by Beau at al. [82].

We performed chemoselective *N*-*trans*-acylation using a reported protocol [82], and converted **63** into the *N*-trifluoroacetyl derivative (NHTFA). This modification relied on direct and chemoselective N-*trans*-acylation by the reaction of **63** with trifluoroacetyl anhydride, allowing an increased solubility, while facilitating the following steps. Next, we produced disaccharide **64** by installing the STolyl (STol) group at the anomeric position, using *p*-methylthiophenol under BF_3_·OEt_2_ conditions, in 80% yield. Methanolysis of **64** and successive protection of 3,4-O positions with the butane-2,3-diacetal moiety and protection of O-6 with TBDMS, using TBDMSCl, TEA, and DMAP in DMF for 16 h afforded the corresponding product. This procedure was executed in 56% yield.

In our first attempts, we were not successful when introducing the lactyl moiety directly into the precursor possessing the NTFA group. Thus, to avoid the random introduction of the lactate moiety, the NTFA group was replaced by the NAc group, by treatment with NaOH in THF at 80 °C, followed by *N*-acetylation with acetic anhydride in pyridine, affording **65** in 56% yield. The lactyl unit was then introduced by treatment of **65** with propionic ester triflate, and **66** was attained in 38% yield. Intermediate **66** possesses all functional groups (*O*-lactyl and NAc) required to prepare the NAG-NAM disaccharide, constituting an excellent precursor of this moiety.

In our strategy, we took advantage of the already established β-1,4 glycosidic bond present in the easily-obtained peracetylated chitobiose **63**, avoiding one of the most challenging steps in the synthesis of PGN fragments. This strategy resulted in a considerable reduction of the number of steps required to obtain a NAG-NAM-containing disaccharide, constituting an atom-economy procedure.

**Scheme 12:**
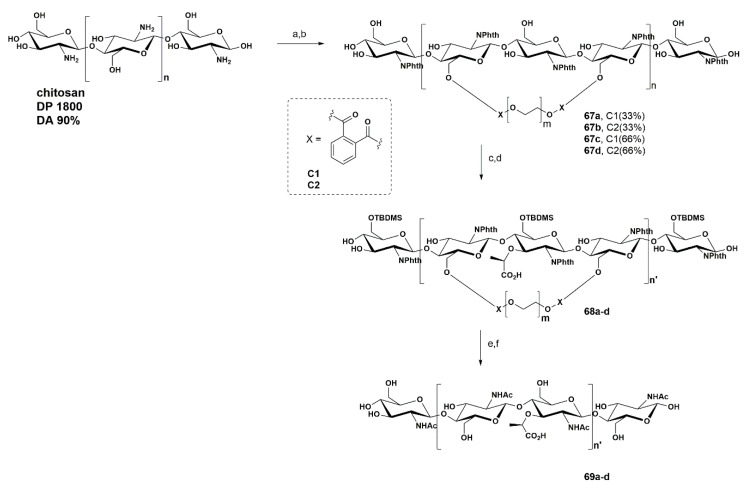
Synthesis of NAG-NAM oligosaccharides (**69**) by Marques group [83]. (**a**) Phthalic anhydride, DMF 5% H_2_O, 120 °C, overnight; (**b**) **C1** (m = 1) or **C2** (m = 2), CDI, DMF, r.t., 24 h; (**c**) TBDMSCl, imidazole, DMF, N_2_, r.t., 72 h; (**d**) (*S*)-2-chloropropionic acid, NaH, DMF, r.t., 48 h; (**e**) (1) NH_2_NH_2_, H_2_O, reflux, 24 h; (2) Ac_2_O, pyridine, r.t., overnight; (**f**) TBAF, pyridine, r.t., overnight.

In the follow-up of our synthesis of an advanced NAG-NAM precursor from chitobiose, we next reported the synthesis of NAG-NAM-containing oligosaccharides from chitosan via a top-down approach involving both chemical and enzymatic reactions (Scheme 12) [83]. This new route was able to overcome the difficulties in obtaining sufficient long fragments to replace samples of biological origin, which also constituted a limitation for further biological investigations [9].

Given the natural resemblance between the carbohydrate backbone of the PGN and chitosan, we envisaged that a properly functionalized chitosan derivative would provide a suitable platform for an alternate attachment of the Lac unit. This new polymer, after removal of the protecting groups, could then be recognized as a PGN substrate by enzymes involved in the degradation of bacterial PGN [84,85,86,87], which would afford the production of smaller oligosaccharides containing NAG-NAM units. PGN hydrolases, such as lysozyme or mutanolysin, can efficiently hydrolyze the β-1,4 glycosidic bond in the natural PGN substrate, between NAM and NAG residues [84,85,86,87,88] and in certain conditions and to some extent in chitin and derivatives [89,90].

We initiated the synthesis by using the phthaloyl group as the N-protecting group of the commercial chitosan, preventing the problems associated with the poor nucleophilicity of NAG derivatives. In order to promote the attachment of the Lac unit in alternate glucosamine units, we explored the use of a molecular clamp strategy, consisting of the formation of a stable bridge between two 6-OH positions of alternate glucosamine units via an ester linkage. Thus, two clamps were synthesized possessing different lengths **C1** (m = 1) and **C2** (m = 2). Different amounts of **C1** and **C2** were tested to maximize the attachment of the Lac moiety in alternate positions. Thus, treatment of the *N*-phthaloyl chitosan with pre-activated acids **C1** or **C2** with CDI in DMF led to the formation of compounds **67**. In order to prevent the introduction of Lac moieties in NAG residues that had not reacted with clamps **C1** and **C2**, compounds **67** were further silylated at the remaining free 6-OH positions by reaction with TBDMSCl in the presence of imidazole, in DMF. Next, we performed the introduction of the Lac moiety, by treatment with NaH in DMF, using (*S*)-(−)2-chloropropionic acid under different conditions and equivalents, affording compounds **68a**–**d**. The sequential removal of the phthalimide group by treatment with hydrazine solution and simultaneous removal of the ester clamp, followed by N-acetylation with acetic anhydride and finally silyl group removal, by treatment with a TBAF solution, afforded compounds **69a**–**d**. Due to the polymeric character of the substrates, the structural characterization was performed by FT-IR and ^13^C CP/MAS NMR (cross polarization magic angle spinning nuclear magnetic resonance).

The ratio of NAM/NAG in samples **69a**–**d** was determined by high performance anion exchange chromatography coupled with pulsed amperometric detection (HPAEC-PAD) and compared with values determined in samples of bacterial PGN purified from *Staphylococcus aureus* and *Escherichia coli* (values of 1.2 and 1, respectively, used as positive controls). We found that the NAM/NAG ratio determined in samples **69a**–**d** was dependent on the amounts of **C1**/**C2** and loadings of the (*S*)-(−)2-chloropropionic acid used in our synthetic procedure. The best results were obtained when a higher loading of C1, together with 2 equiv. of (*S*)-(−)2-chloropropionic acid, were used. The sample **69** produced in these conditions had a NAM/NAG ratio of 1.21, similar to that found in bacterial PGN purified from *Staphylococcus aureus* bacteria.

Having confirmed that our synthetic procedures were able to result in a significant incorporation of NAM residues on the chitosan skeleton, indicated by the ratio of NAM/NAG, we next investigated whether alternated NAM units had been installed. This was performed by using PGN lytic enzymes, such as mutanolysin and lysozyme, and by analysis of the fragments released by LC-MS. We found that 55% of the digested **69** generated the disaccharide NAG-NAM in 7% yield, as well as the trisaccharide NAG-NAG-NAM in 33% yield.

This work highlights that the combination of chemical and chemical/enzymatic approaches is an extremely attractive option, compared to traditional orthogonal synthesis, in the synthesis of complex and biological important molecules. Of note, several enzymatic strategies, which use particular purified bacterial enzymes for the production of PGN precursors/fragments, have also been recently developed [91,92,93].

## 5. Conclusions

PGN is a major constituent of the bacterial cell wall. Although its biosynthesis is assisted by several enzymes that sequentially transform specific sugar precursors and assemble them into the complex structure of the natural PGN, its chemical synthesis poses several problems. Despite all efforts developed by us and others, the synthesis of PGN fragments remains a challenge. The key difficulty is based on stereoselective glycosylation involving NAG derivatives. Indeed, the complex synthesis of the carbohydrate backbone commonly involves multistep sequences to install the lactyl moiety at the O-3 position and to control enantioselective glycosylation.

This review presents the biosynthetic pathways, as well as covering several approaches used to assemble the complex PGN fragments, including one-pot regioselective protection, orthogonal approaches, and the recently reported chemoenzymatic route towards key intermediates to achieve NAG-NAM moieties.

The synthesis of bacterial PGN fragments is crucial in order to better understand the role of this macromolecule in the metabolism of bacteria and their recognition by different hosts. This review collects the approaches, the difficulties imposed by the natural PGN structure, and the discoveries made by the community to address the synthesis of PGN molecules.
